# When a sore throat turns into deadly multiple serous cavity effusions: the role of *Prevotella oris* in rapidly progressing infection—a case report

**DOI:** 10.3389/fmed.2025.1517389

**Published:** 2025-01-29

**Authors:** Fangqi Zhang, Juan-Li Wang, Jian Zhu, Shaokui Si, Hao Guo, Xiang Yue, Wei Wen

**Affiliations:** ^1^Department of Pulmonary and Critical Care Medicine, The 987th Hospital of Joint Logistics Support Force of People's Liberation Army, Baoji, China; ^2^Department of Child Healthcare, The Third Hospital of Wuhan, Wuhan, China; ^3^Department of Thyroid and Breast Surgery, General Hospital of Central Theater Command of the People's Liberation Army, Wuhan, China; ^4^Department of Thoracic Cardiovascular Surgery, The 987th Hospital of Joint Logistics Support Force of People's Liberation Army, Baoji, China; ^5^Department of Thoracic Surgery, The First Affiliated Hospital with Nanjing Medical University, Nanjing, China

**Keywords:** polyserous effusions, *Prevotella*, sore throat, necrotizing mediastinitis, case report

## Abstract

Severe infections that develop rapidly from ordinary symptoms not only increase patient misunderstandings but also lead to excessive detection of these symptoms by physicians. This case study describes a 19-year-old male individual who initially presented with a sore throat and subsequently developed multiple serous cavity effusions that lead to septic pulmonary embolism and septic shock. After multiple cultures of the patient’s sputum yielded no identifiable pathogenic bacteria, the metagenomic next-generation sequencing (mNGS) revealed *Prevotella oris* as the predominant pathogen present in both the patient’s peripheral blood and the pericardial drainage fluid. The subsequent antibiotic treatment, guided by the mNGS results, along with surgical drainage and mediastinal irrigation, effectively controlled and ultimately cured the patient’s condition. This case is unique because it is the first to show that normally colonizing *Prevotella* can also cause fatal multiorgan infection as an opportunistic pathogen in a previously healthy young person with no immune-related diseases. The aim of this study is to expand clinical awareness of this common symptom and its potentially fatal outcome.

## Introduction

*Prevotella* is often considered part of the Bacteroides complex, commonly associated with a healthy plant-based diet, and acts as a probiotic in the human body, throughout the entire digestive tract, from the mouth to the anus (1, 2). It can survive in atmospheric oxygen concentrations for up to 3 days (3).

Previous reports have shown that *Prevotella* species can lead to pleural infection (4, 5). However, there are no published reports discussing fatal multiple serous cavity effusions caused by *Prevotella oris* in a previously healthy young person with no immune-related diseases. This case study presents a 19-year-old male who initially presented with a sore throat and subsequently developed multiple serous cavity effusions, which led to septic pulmonary embolism and septic shock caused by *Prevotella oris.*

## Case report

**Figure 1:**
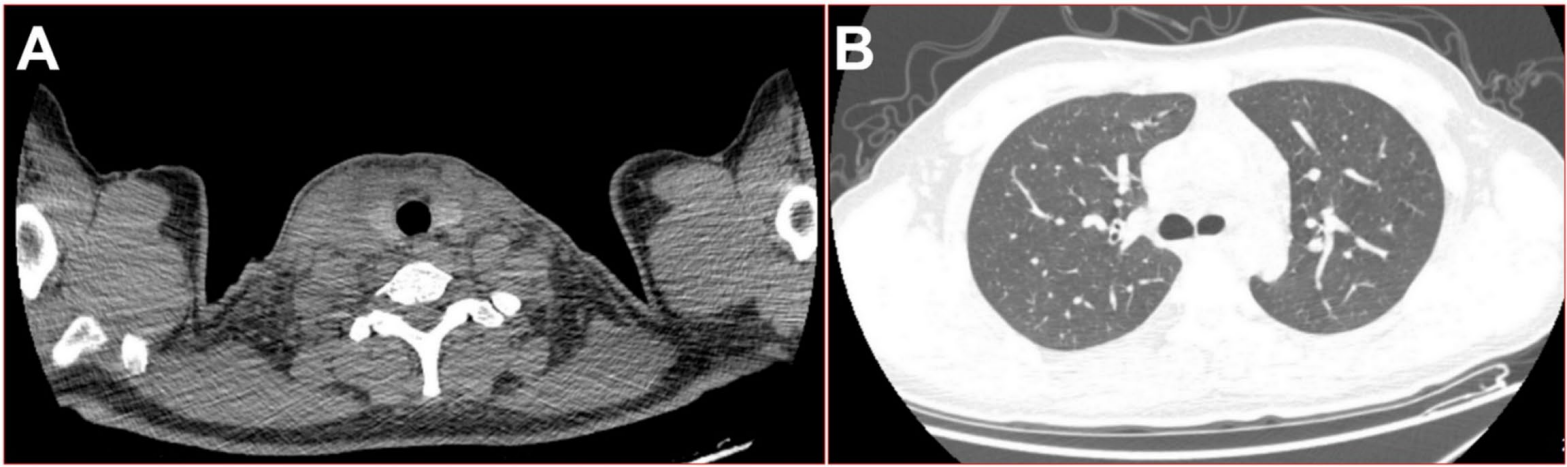
On the fourth day of the fever, 16-slice computed tomography (CT) images of the patient were obtained. **(A)** Swelling of the right side of the neck soft tissue; **(B)** lung window in the upper thoracic cavity.

**Figure 2:**
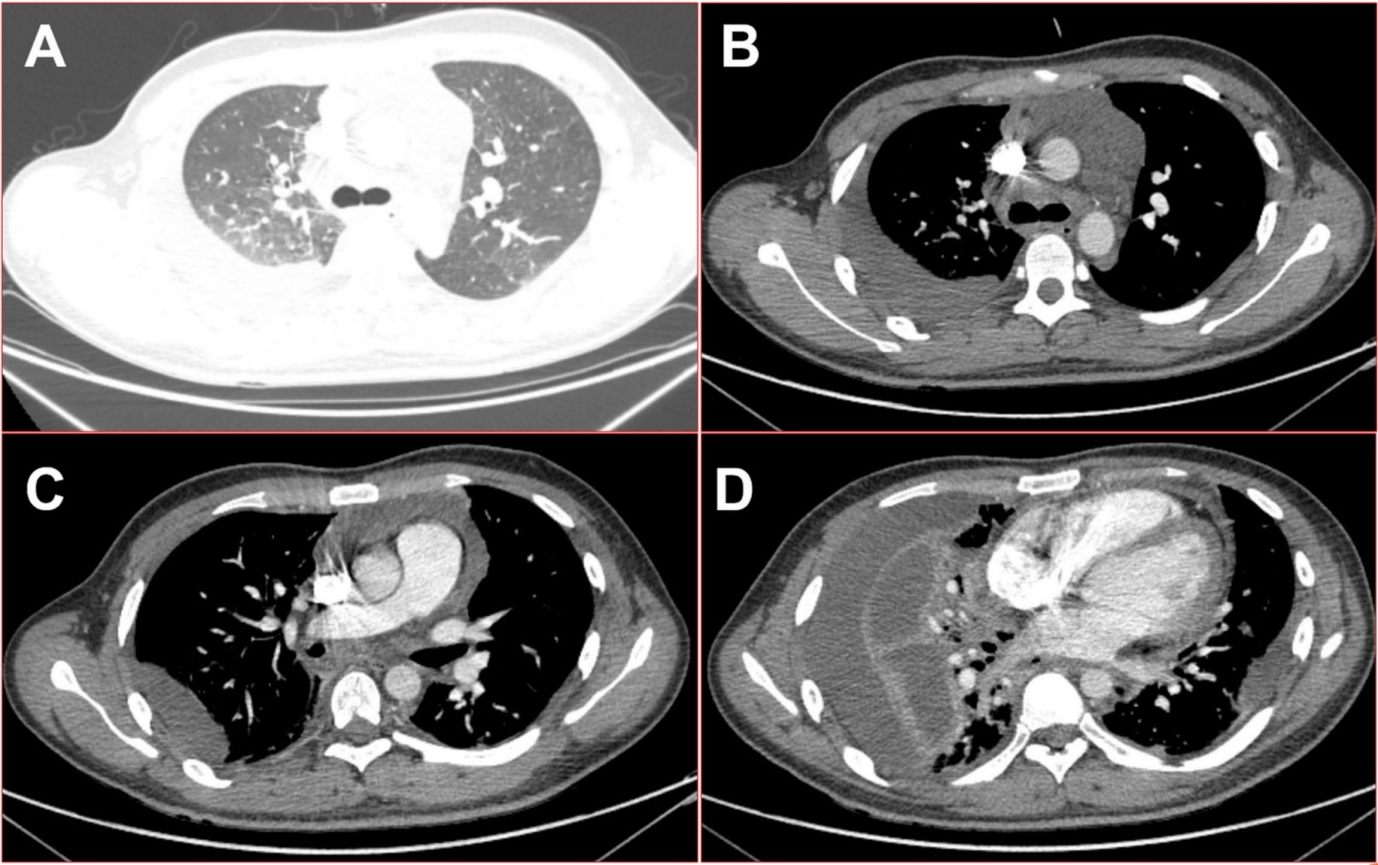
On the seventh day of the fever, 16-slice computed tomography images of the patient’s chest revealed multiple serous cavity effusions. **(A)** Lung window in the upper thoracic cavity; **(B)** mediation window in the upper thoracic cavity; **(C)** mediation window in the middle part of the thoracic cavity; **(D)** mediation window in the lower thoracic cavity.

A 19-year-old male automobile manufacturing worker, previously healthy with no history of immune-related diseases, presented with a sore throat and fever lasting 1 day, with a maximum temperature of 38.9°C. He had neither a history of specific infectious diseases nor any exposure to pollen or pets. He denied a family history of hereditary diseases. He was diagnosed with tonsillitis at a community hospital and received cefixime treatment for 3 days. However, his condition did not improve, and he developed swelling on the right side of his neck. The computed tomography (CT) examination revealed no obvious abnormalities in the lungs or the chest cavity but showed suspicious swelling of the soft tissue on the right side of the neck (see Figure 1). The hemograms revealed a leukocyte count of 16.86 × 10^9^/L (normal range: 3.5–9.5 × 10^9^/L) and a neutrophil count of 93.6%. Based on these results, he was treated with intravenous moxifloxacin. Two days later, the above-mentioned symptoms continued to worsen, and he developed chest pain and shortness of breath. He was then admitted to the hospital. When asked about his recent lifestyle habits, he denied drinking probiotic drinks or consuming functional dairy products. He also denied eating raw or unclean food, including during the period of antibiotic treatment. The physical examination revealed an acute disease presentation, with pharyngeal congestion, bilateral tonsil enlargement (grade II), evident neck swelling, rapid breathing (45 breaths per min), audible wet rales in both upper lungs, diminished respiratory sounds in both lower lungs, a heart rate of 134 beats per min, a consistent rhythm, distant heart sounds, and abdominal tenderness. The dental examination revealed mild swelling of his gums, no decayed teeth, and good oral hygiene. He underwent a chest CT re-examination, which showed a large amount of fluid in the chest, pericardial, and abdominal cavities. The right lung was infected by a scattered pattern (see Figure 2). The microbiological testing of the pharyngeal swab did not detect the flu, coronavirus, or any bacterial microorganisms. The laboratory tests, including HIV tests and T-SPOT-TB tests, were negative. The hemograms revealed a leukocyte count of 25.74 × 10^9^/L, with a neutrophil percentage (NEUT%) of 92.1%. Other results included a C-reactive protein level of 265.76 mg/L, procalcitonin of 4.2 ng/mL, albumin of 23.2 g/L, and the following arterial blood gas values (with 5 L/min oxygen mask): pH 7.39, PO2 80 mmHg, PCO2 36 mmHg, and lactate (Lac) 2.3 mmol/L. Therefore, he underwent emergency tracheal intubation and ventilator-assisted breathing, and his treatment regimen was changed to a combination of meropenem and linezolid as broad-spectrum antibiotics for severe sepsis.

**Figure 3:**
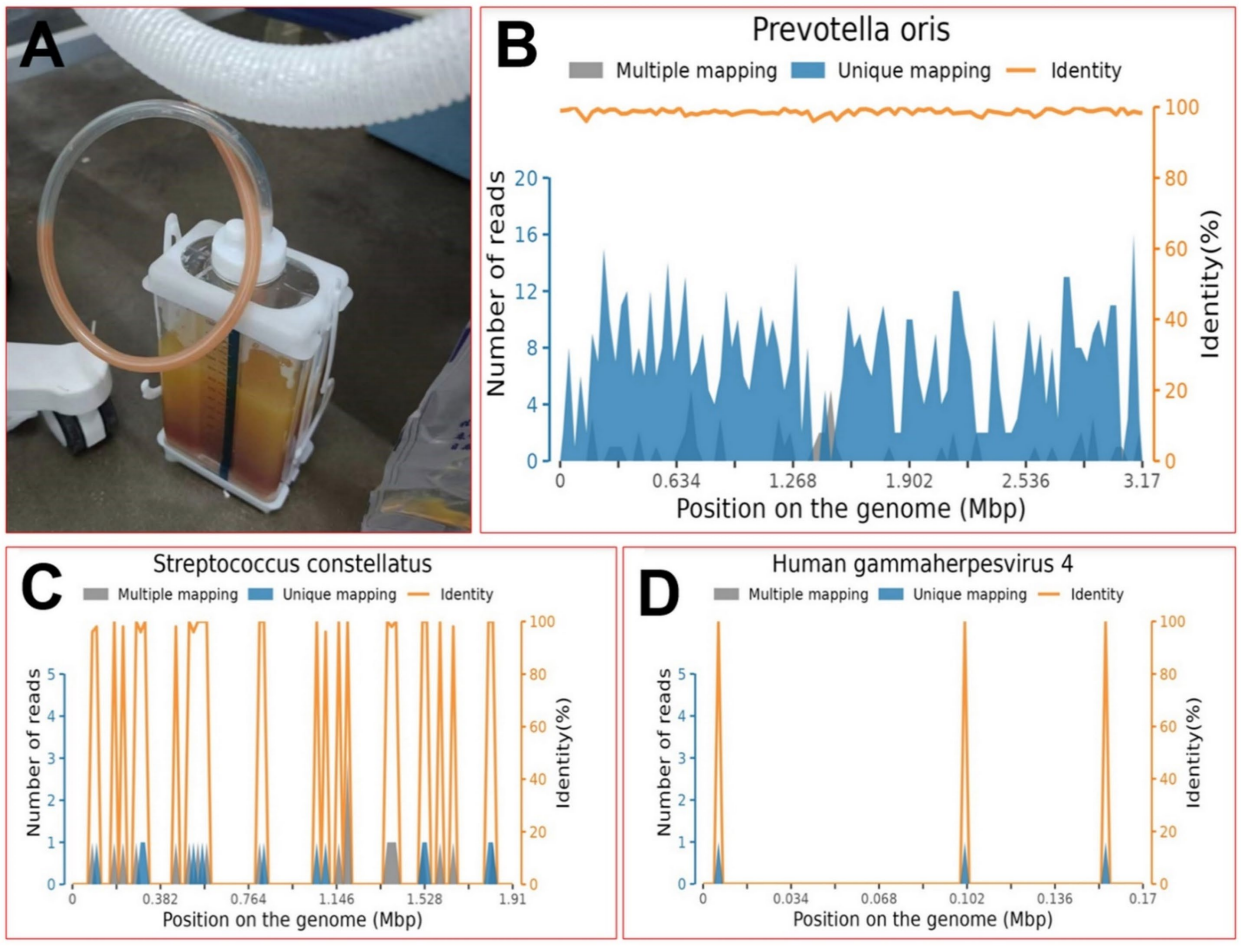
On the seventh day of the fever, the diagnosis was determined. **(A)** Appearance of pleural effusion; **(B)** position map of *P. oleracea* via metagenomic next-generation sequencing; **(C)** position map of *S. constellatus* via metagenomic next-generation sequencing; **(D)** position map of *HGM4* via metagenomic next-generation sequencing.

**Figure 4:**
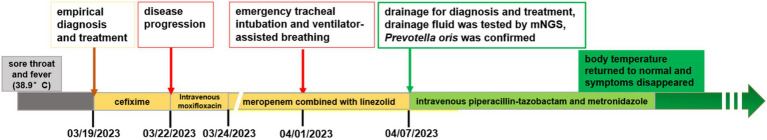
A figure explicitly showcasing the timeline with clinical and diagnostic milestones in this case.

Then, he underwent closed thoracic drainage for diagnosis and treatment, during which 1,000 mL of red and white, gradually stratified, turbid fluid was removed, emitting a foul odor. During this period, the patient was repeatedly cultured for bacteria, tuberculosis, and fungi in the pus, blood, and sputum collected from the breathing tube, and no positive findings were observed. The biochemical examination of the pleural effusion showed a WBC count of 172.11 × 10^9^/L, a NEUT percentage of 91.0%, and a RBC count of (2+). Pericardial puncture fluid was also obtained, and the thoracic cavity, pericardial drainage fluid, and peripheral blood were examined for pathogenic bacteria using metagenomic next-generation sequencing (mNGS), which identified a large number of *Prevotella oris* (see Figure 3). A figure explicitly showcasing this timeline with clinical and diagnostic milestones is presented in Figure 4.

**Figure 5:**
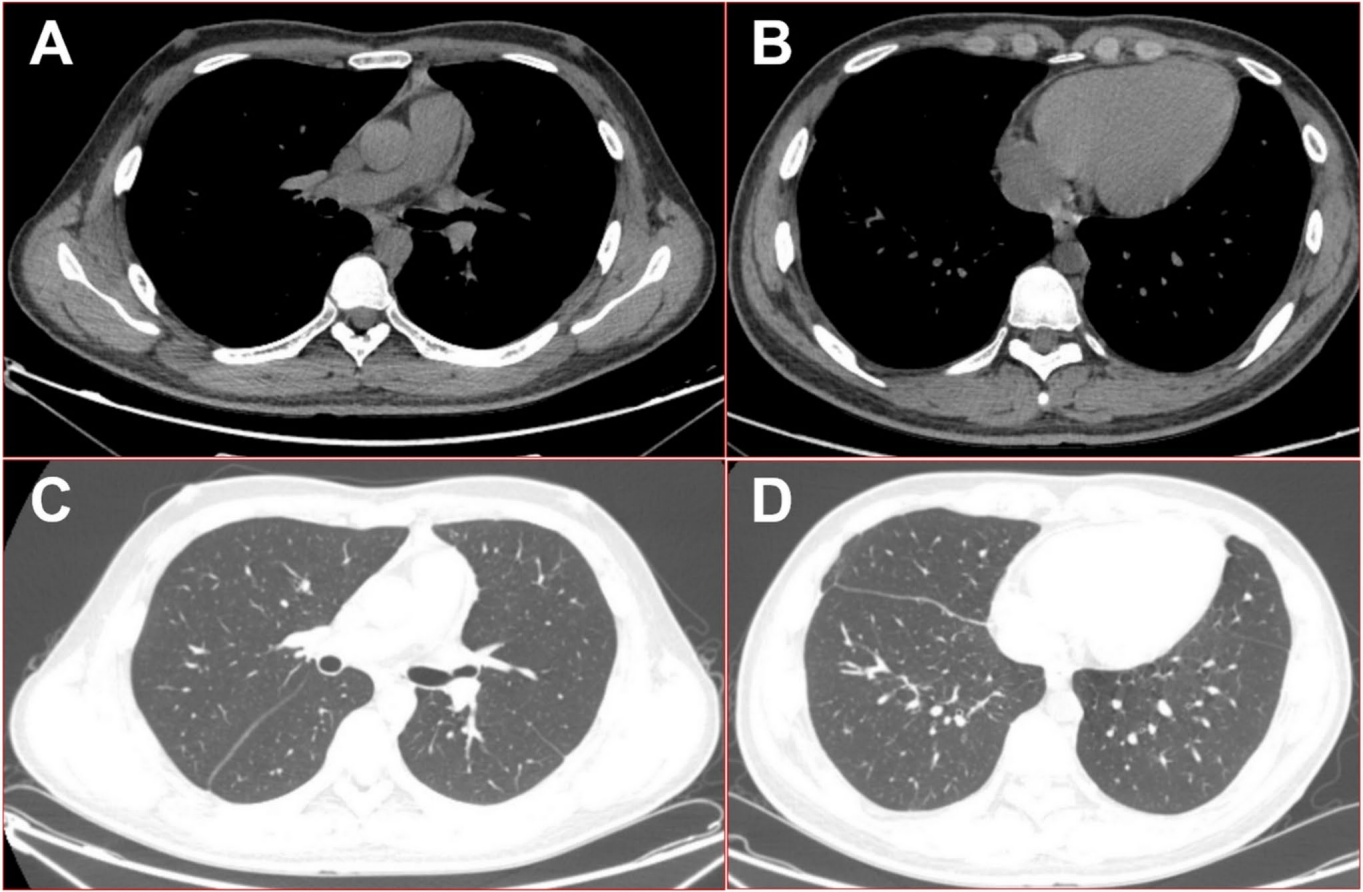
16-slice computed tomography images of the chest were obtained during the follow-up after the patient was cured. **(A)** Mediation window in the upper thoracic cavity; **(B)** mediation window in the lower thoracic cavity; **(C)** lung window in the upper thoracic cavity; **(D)** lung window in the lower thoracic cavity.

Subsequent antibiotic treatment, guided by the mNGS results, included intravenous piperacillin-tazobactam and metronidazole, along with aggressive surgical flushing and continuous drainage of the multilocular cavity. Within a few days, the patient made a rapid recovery, with a return to normal body temperature, resolution of respiratory symptoms, and quick healing of the drain site. Three months after discharge, the patient’s CT re-examination showed that the lesion had mostly resolved (see Figure 5), and he expressed great satisfaction with the diagnosis and treatment he received during his hospital stay.

## Discussion

The patient was a young adult with a healthy immune system. The main complaint was pharyngeal pain accompanied by fever, with the pain persisting throughout the course of the disease. The patient’s condition rapidly progressed from neck symptoms (right-side neck swelling) to chest symptoms (pleural and pericardial effusions), eventually leading to fatal multiple serous cavity effusions. Factors that contributed to the progression to sepsis with *Prevotella oris* included the lack of positive results from the pathogen cultures and insufficient knowledge about infections caused by *Prevotella oris*. The key to obtaining an accurate diagnosis is recognizing that the patient has a typical persistent fever and the evidence of elevated white blood cells. Moreover, rapid progression of the disease is not characteristic of tuberculous multiple serous cavity effusions. The patient was treated with a broad spectrum of antibiotics, and although the disease continued to progress, no positive findings were observed in the pus, blood, or sputum pathogen cultures. This indicates that the likelihood of detecting *Prevotella oris* in the blood or sputum pathogen cultures was low.

The presence of a red and white stratified turbid liquid suggested the possibility of chylous fluid. However, chylous fluid rarely causes symptoms of infection. Furthermore, there are three main causes of chylous leakage: (1) trauma, such as closed or open injuries to the neck and chest (6); (2) obstruction, such as lymphoma, metastatic cancer, or mediastinal granuloma (7); and (3) congenital thoracic duct hypoplasia or fistula formation (8). Other causes include tuberculosis, cirrhosis, sarcoidosis, and lymphatic malformations (9). The patient in this case had no history of neck trauma. Obstruction and congenital thoracic duct hypoplasia or fistula formation often occur in the abdomen, primarily affecting children. The symptoms and signs presented in this case were not consistent with the aforementioned etiologies or the disease characteristics of chylous fluid.

After escalating the empirical broad-spectrum antibiotic treatment, the patient continued to progress, and septic shock developed. Meanwhile, the patient’s sputum drawn from the breathing tube was cultured repeatedly, but no obvious positive pathogenic bacteria were found. Fortunately, *Prevotella oris* was accurately detected as the pathogen using mNGS, which is theoretically able to detect any pathogen with a known genome sequence (10).

*Prevotella* is a genus of obligate anaerobic Bacteroides that colonize the human body extensively by binding to or attaching to other bacteria, rather than epithelial cells (1, 2, 11). *Prevotella* has a natural antibiotic resistance gene that prevents its elimination (12). When abundant, it is beneficial to human health and acts as a probiotic that can break down proteins and carbohydrates (1, 2, 13). It is even sold as an important ingredient in health drinks in some regions. However, this patient denied drinking probiotic drinks or consuming functional dairy products, and he also denied eating raw or unclean food. According to previous literature, the high abundance of this genus may be associated with diseases such as infections and hypertension (14, 15).

*Prevotella oris,* a species of the *Prevotella* genus, is commonly found in the mouth and causes periodontitis, but there have been few reports of it outside the oral cavity. *Prevotella* species, particularly *Prevotella intermedia* and *Prevotella denticola*, are notable for their role as opportunistic pathogens in various diseases, especially periodontitis. These Gram-negative anaerobic bacteria possess several virulence factors that enable them to adhere to host tissues, evade immune responses, and disrupt normal physiological processes. One of the primary virulence factors of *Prevotella* spp. is their ability to produce enzymes, such as cysteine proteases, which play a significant role in tissue degradation and immune evasion. These enzymes can degrade host proteins, thereby facilitating bacterial invasion and persistence in inflamed tissues. In addition, *Prevotella* species express adhesins that enhance their ability to bind to host cells, promoting colonization and biofilm formation. The interaction between *Prevotella* and other oral bacteria, such as *Streptococcus mutans*, can further enhance their virulence by creating a synergistic environment that supports the development of hypervirulent biofilms, which are more resistant to antimicrobial treatments (16, 17). The expression of virulence factors in *Prevotella* spp. is influenced by various environmental conditions, including the presence of host-derived signals and the local microenvironment within the oral cavity or inflamed tissues. For instance, the inflammatory milieu, characterized by elevated levels of cytokines and other immune mediators, can upregulate the expression of virulence genes in these bacteria. This response is critical during periods of dysbiosis, where the balance of microbial communities is disrupted, allowing *Prevotella* to thrive and contribute to disease progression (18). Furthermore, fatal multiple serous cavity effusions caused by *Prevotella oris* have not been reported.

On a microscopic level, *Prevotella oris* in the oropharynx can enter the lung microbiota through trace aspiration (19). Based on the theory of the gut–lung axis, *Prevoiella* in the gut may connect to and colonize the lung flora through the gut–lung axis and other pathways, resulting in similar trends of colonization by *Prevotella* in both the gut and lungs, as well as the crossover of dominant microbial communities (20). Research has indicated that the gut microbiota can produce various metabolites that enter the bloodstream and reach the lungs, where they can modulate immune responses. Short-chain fatty acids (SCFAs), for example, are produced by gut bacteria and have been shown to exert anti-inflammatory effects in the lungs. This underscores the potential therapeutic role of probiotics and prebiotics in managing respiratory diseases by restoring gut microbiota balance and enhancing gut–lung axis communication (20). Furthermore, the interplay between gut microbiota and lung health is not limited to inflammation; it also encompasses the modulation of immune cell activity and the regulation of systemic inflammation, both of which are crucial for preventing respiratory diseases.

In clinical practice, the accepted route of infection is through the oropharynx, which includes the submandibular space, parapharyngeal space, and retropharyngeal space, and is composed of the superficial, middle, and deep layers of the deep cervical fascia (21). Therefore, as in this case, *Prevotella* was likely the cause of the oropharyngeal infection that spread into these spaces and the mediastinum under centripetal force, causing pleural and pericardial effusions and subsequent hypoproteinaemia, which led to abdominal effusion and systemic polyscreening.

Early detection of pathogenic bacteria and multidisciplinary team discussions are essential for rapidly progressing multiple serous cavity effusions infections to obtain targeted treatment and avoid adverse prognoses (22). Detecting *Prevotella* species, particularly in clinical and environmental contexts, requires a nuanced understanding of their properties as anaerobic, Gram-negative bacteria. Their detection can be challenging due to their fastidious nature and the limitations of traditional culture methods. One effective approach for detecting *Prevotella* spp. involves the use of molecular techniques such as quantitative polymerase chain reaction (qPCR). This method has been utilized to develop species-specific primers that enhance the sensitivity and specificity of detection. For instance, a study designed specific primers based on the nucleotide sequence of a previously cloned DNA probe, which allowed for the accurate identification of *Prevotella nigrescens* in clinical samples (23). Furthermore, the application of metagenomic next-generation sequencing (mNGS) has proven beneficial in identifying rare pathogens such as *Prevotella intermedia*, which was detected in cerebrospinal fluid, showcasing the potential of advanced sequencing technologies in clinical diagnostics (24). In addition to molecular methods, the use of selective culture media can significantly improve the recovery of *Prevotella* species from clinical specimens. A systematic screening of various microbiological media has been shown to enrich for previously uncultivated target species associated with periodontal health or disease (25). This approach not only aids in isolating these bacteria but also provides insights into their ecological roles within the microbiome. Moreover, the dynamics of *Prevotella* spp. in the human gut microbiota have been extensively studied, revealing their complex interactions with other microbial communities and their implications for health. For example, a longitudinal study highlighted the transient nature of *Prevotella* abundance, suggesting that environmental factors and dietary habits can influence their detection and prevalence in the gut (26). This underscores the importance of considering both the biological and environmental contexts when developing detection strategies for *Prevotella* spp. Adequate clinical symptoms, signs, and medical imaging are also essential (4, 27, 28).

In summary, this case study exemplifies the complex journey of a physician’s diagnosis and treatment of a previously healthy young man who experienced the progression of an infection, from an oral infection to multiple serous cavity effusions. Through this intricate process, the detrimental effects of *Prevotella oris* as a prevalent probiotic pathogen are highlighted, emphasizing its potential for disastrous consequences. This case study offers valuable insights into understanding a category of infectious diseases characterized by typical clinical symptoms but with severe outcomes, ultimately aiming to mitigate medical disputes and enhance the management of rare diseases.

## Data Availability

The original contributions presented in the study are included in the article/supplementary material, further inquiries can be directed to the corresponding authors.
